# Structure basis 1/2SLPI and porcine pancreas trypsin interaction

**DOI:** 10.1107/S090904951302133X

**Published:** 2013-09-29

**Authors:** Kei Fukushima, Takashi Kamimura, Midori Takimoto-Kamimura

**Affiliations:** a Medicinal Chemistry Technology Department, Teijin Institute for Bio-Medical Research, 4-3-2 Asahigaoka, Hino-shi, Tokyo 191-8512, Japan

**Keywords:** serine protease, protein complexes, protein crystallization, protein structure, X-ray crystallography, enzyme inhibitors, SLPI

## Abstract

SLPI (secretory leukocyte protease inhibitor) is a 107-residue protease inhibitor which inhibits various serine proteases, including elastase, cathepsin G, chymotrypsin and trypsin. SLPI is obtained as a multiple inhibitor in lung defense and in chronic airway infection. X-ray crystal structures have so far reported that they are full-length SLPIs with bovine α-chymotrypsin and 1/2SLPI (recombinant C-terminal domain of SLPI; Arg58–Ala107) with HNE (human neutrophil elastase). To understand the role of this multiple inhibitory mechanism, the crystal structure of 1/2SLPI with porcine pancreas trypsin was solved and the binding modes of two other complexes compared. The Leu residue surprisingly interacts with the S1 site of trypsin, as with chymotrypsin and elastase. The inhibitory mechanism of 1/2SLPI using the wide primary binding site contacts (from P2′ to P5) with various serine proteases is discussed. These inhibitory mechanisms have been acquired in the evolution of the protection system for acute inflammatory diseases.

## Abstract 2

1/2SLPI is a C-terminal domain of SLPI (secretory leukocyte protease inhibitor) which inhibits various serine proteases broadly. The present study is the first X-ray structural report on how 1/2SLPI with P1 Leu strongly inhibits trypsin and how it can inhibit multiple serine proteases.

## 1. Introduction

Secretory leukocyte protease inhibitor (SLPI) is a non-glycosylated serine protease inhibitor of 107 amino acids. It inhibits various serine proteases, including elastase, cathepsin G, chymotrypsin and trypsin (Seemüller *et al.*, 1986 ▶; Smith & Johnson, 1985 ▶; Thompson & Ohlsson, 1986 ▶). SLPI has been found in a variety of fluids, including saliva (Ohlsson *et al.*, 1983 ▶), bronchi mucus (Ohlsson *et al.*, 1977 ▶), tears (Kueppers, 1971 ▶), cervical mucus (Wallner & Fritz, 1974 ▶) and seminal plasma (Schiessler *et al.*, 1976 ▶). In the respiratory tract, SLPI is produced by serous cells of tracheal and bronchial submucosal glands and by non-ciliated bronchiolar epithelial cells, identified as goblet and clara cells (Kramps *et al.*, 1981 ▶, 1989 ▶; Willems *et al.*, 1986 ▶). The exact physiological function of SLPI has not been elucidated, but its major role is considered to be the protection of the airway epithelial surface from attached human neutrophil elastase which has been found in pulmonary lavage fluid and sputum from patients with inflammatory respiratory diseases (Scott *et al.*, 2011 ▶; Zani *et al.*, 2011 ▶). Recently, in addition to the serine protease inhibitory activity, antimicrobial and anti-HIV activities (Shine *et al.*, 2002 ▶) have been reported to be associated with inflammations that have been shown to promote wound healing in the skin and other non-neural tissues. SLPI has an early protective effect after central nervous system injury and reduces secondary tissue damage by suppression of NFκB (Clauss *et al.*, 2002 ▶; Ghasemlou *et al.*, 2010 ▶; Taggart *et al.*, 2005 ▶). Structurally, SLPI has two non-hydrophobic core domains and contains typical secondary structures in both N- and C-terminal domains. 1/2SLPI (recombinant C-terminal domain of SLPI Arg58–Ala107) has a potent inhibitory activity against multiple serine proteases such as trypsin, chymotrypsin, elastase, chymase and cathepsin G as that of intact SLPI (Korkmaz *et al.*, 2010 ▶; Masuda *et al.*, 1995 ▶; Rao *et al.*, 1993 ▶). In particular, 1/2SLPI showed quite strong anti-human neutrophil elastase activity. In terms of X-ray structural analysis, there are reports of the 1/2SLPI–HNE complex (HNE: human neutrophil elastase) (Koizumi *et al.*, 2008 ▶) and SLPI–chymotrypsin complex structures [atomic coordinates kindly provided by Dr W. Bode (Grütter *et al.*, 1988 ▶)]. Generally, a main part of the affinity and inhibitory activity of the biological inhibitors depends on the P1 residue which penetrates deeply into the S1 pocket of the proteases (Otlewski *et al.*, 2001 ▶). SLPI had a strong affinity with the S1 site by the P1 Leu72i residue and inhibits chymotrypsin in the structural reports. Additionally, 1/2SLPI also inhibits porcine pancreatic trypsin concentration at about 140 n*M* and we succeeded in obtaining the 1/2SLPI and trypsin complex. The present work describes the first X-ray structure analyses of 1/2SLPI with P1 Leu residue with trypsin (PPT) at 2.0 Å and comparisons are made with the previously reported 1/2SLPI–HNE and SLPI–chymotrypsin complex structures. We also discuss the inhibitory mechanism of 1/2SLPI against various serine proteases.

## 2. Materials and methods

### 2.1. Protein expression and purification of 1/2SLPI

Expression and purification of 1/2SLPI were essentially carried out as described in our previous reports (Masuda *et al.*, 1992 ▶, 1996 ▶). The synthetic fragment of the SLPI gene encoding residues Arg58–Ala107 was cloned into pUC119 (Takara Shuzo Co.) with a thrombin cleavage site at the N-terminus. The resulting plasmid was transformed into *E. coli* HB101, and the transformed cells were cultured followed by collection of inclusion bodies, thrombin treatment, intramolecular disulfide refolding and purification by chromatography.

### 2.2. Measurement of the protease inhibitory activity of 1/2SLPI

*For HNE and PPE (porcine pancreas elastase).* The substrate was MeO-Suc-Ala-Ala-Pro-Val-pNA (Bachem Holding AG) for HNE (Elastin Products Company) and PPE (Wako-chemical) and the reaction buffer used for both enzymes was 100 m*M* HEPES–NaOH (pH 7.5) containing 1.0 *M* NaCl, 0.1% (*w*/*v*) PEG6000. The required inhibitor solution was pre-incubated for 1 h at 298 K with an appropriate amount of the required enzyme for 5 n*M* HNE or 10 n*M* PPE. The reaction was started by adding the substrate and carried out for 20 min at 298 K. The absorbances of the reaction mixture at 405 nm were recorded and the inhibitory constants were determined (Henderson, 1972 ▶; Masuda *et al.*, 1995 ▶).

*For BPT (bovine pancreas trypsin) and PPT (porcine pancreas trypsin).* The substrate was pyro-Glu-Gly-Arg-pNA (S-2444; Paar & Marhuln, 1980 ▶) (Dia Pharma Group) for BPT (Sigma-Aldrich) and PPT (Sigma-Aldrich). The reaction buffer used for both enzymes was 50 m*M* Tris-HCl (pH 8.0), 100 m*M* NaCl, 0.01% (*w*/*v*) Tween20. The required inhibitor solution was pre-incubated for 10 min at 298 K with an appropriate amount of the required enzyme for 0.5 n*M* BPT or 0.2 n*M* PPT. The absorbance of the reaction mixture at 405 nm was recorded and the inhibition constants were determined (Henderson, 1972 ▶; Masuda *et al.*, 1995 ▶).

*For bovine α-chymotrypsin.* The substrate was MeO-Suc-Ala-Ala-Pro-Phe-pNA (Bachem Holding AG) and the reaction buffer used for the enzyme was 100 m*M* HEPES–NaOH (pH 7.5), containing 1.0 *M* NaCl, 0.1% (*w*/*v*) PEG6000. The required inhibitor solution was pre-incubated for 1 h at 298 K with an appropriate amount of the required enzyme for 30 ng ml^−1^ chymotrypsin (Sigma-Aldrich). The absorbance of the reaction mixture at 405 nm was recorded and the inhibition constants were determined (Henderson, 1972 ▶; Masuda *et al.*, 1995 ▶).

### 2.3. Crystallization and structure determination

The purified 1/2SLPI and PPT were mixed with a 1:1 molar ratio in 50 m*M* Tris-HCl (pH 7.5) and incubated for 2 h at room temperature. The following sample was dialyzed against 50 m*M* Tris-HCl (pH 7.5) and concentrated to 30 mg ml^−1^ as PPT and 1/2SLPI complex. The initial crystallization screening was performed by the sitting-drop vapour-diffusion method at 293 K, using Jena Bioscience Screening kits. The appropriate crystals were obtained from 25% PEG4000, 100 m*M* Na-citrate (pH 5.6), 200 m*M* ammonium sulfate. X-ray diffraction data were collected by FR-E (Rigaku) from a single frozen crystal. The collected data were processed with *CrystalClear* version 1.3.5 and solved by the molecular replacement method using the CCP4 version 4.2 suite with the X-ray structure of 1/2SLPI [Protein Data Bank (PDB) code: 2z7f; Koizumi *et al.*, 2008 ▶] and the structure of PPT (PDB code: 1avw; Song & Suh, 1998 ▶) as search models. Structure refinements were performed with *REFMAC5*. The quality of the final structures was accessed with *PROCHECK* prior to depositing at the PDB under the code 4doq. The detailed data-collection statistics are presented in Table 1 ▶.

## 3. Results and discussion

The inhibitor constant (*K*_i_) values of 1/2SLPI against the target serine proteases, HNE, PPE, chymotrypsin, BPT and PPT, are shown in Table 2 ▶. 1/2SLPI strongly inhibits not only HNE and chymotrypsin, but also PPT with *K*_i_ = 140 n*M* and BPT with *K*_i_ = 36 n*M*. Our biological assay result shows that 1/2SLPI strongly inhibits various serine proteases broadly. Many serine proteases and their intact proteinase inhibitors like SLPI, BPTI and OMTKY3 have been analyzed such that the interaction between the P1 residue and the S1 pocket is most important according to various mutation studies (Baranger *et al.*, 2011 ▶; Bode *et al.*, 1986 ▶; Eisenberg *et al.*, 1990 ▶; Hanson *et al.*, 2007 ▶; Helland *et al.*, 1999 ▶; Kawamura *et al.*, 2011 ▶; Masuda *et al.*, 1994 ▶; Qasim *et al.*, 2006 ▶; Zani *et al.*, 2009 ▶). Serine proteases can be further categorized based on their substrate specificity as either trypsin-like or chymotrypsin-like. In the inhibition against trypsin-like serine proteases, the P1 residue generally favored a positively charged residue like Arg or Lys which can tightly interact with a negatively charged residue like Asp commonly located at the bottom of the S1 pocket. On the other hand, hydrophobic P1 residues like Leu favored chymotrypsin-like serine protease. Eisenberg *et al.* (1990 ▶) show that the replacement of the P1 residue of full-SLPI from Leu72i to Arg72i drastically increases the inhibitory activity against trypsin from *K*_i_ = 3.0 n*M* to *K*_i_ < 0.001 n*M*, but the replacement from Leu72i to Phe72i also increases the inhibitory activity from 3 n*M* to 0.3 n*M*. These results show that the hydrophobic P1 residues are also acceptable, like the positively ionized residues for the inhibition against trypsin. Therefore, it is consistent that P1 Leu72i of 1/2SLPI is used to inhibit trypsin. It has not been shown in the OMTKY3 (Qasim *et al.*, 2006 ▶) and BPTI variants (Hanson *et al.*, 2007 ▶; Helland *et al.*, 1999 ▶) that Leu is used as a P1 residue against trypsin.

**Figure 1:**
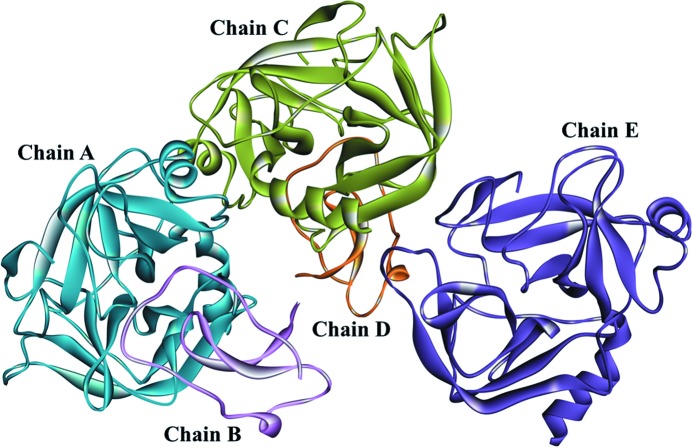
Structure of the 1/2SLPI–porcine pancreas β-trypsin complex in an asymmetric unit.

The structure of the 1/2SLPI and PPT complex was refined by usual methods and the experimental data and refinement statistics are summarized in Table 1 ▶. The complex crystal involved two 1/2SLPI (between Lys60i and Lys106i residue; chains B and D) and three PPT (between Ile16 and Asn245 residue; chains A, C and E) molecules per asymmetric unit as shown in Fig. 1 ▶. Two complexes (one with chains A and B, the other with chains C and D) appear to interact with the reversible inhibition mechanism. Additionally, one uncomplexed PPT molecule (chain E) exists per asymmetric unit. The main-chain structures of two 1/2SLPI and three PPT are almost identical. Two independent 1/2SLPI molecules are superposing each other at the corresponding main-chain (Lys60i–Lys106i) atoms and the root-mean-square difference (RMSD) is 0.19 Å. The RMSD value among three independent PPT molecules was 0.35 Å by superposing with the corresponding main-chain atoms between residues Ile16 and Asn245. In comparison with the other X-ray structural analysis, the 1/2SLPI molecules observed here are quite similar to the 1/2SLPI structures already reported which complexed with HNE (PDB code: 2z7f; Koizumi *et al.*, 2008 ▶) and with chymotrypsin (Grütter *et al.*, 1988 ▶). RMSD values from the corresponding main-chain atoms of 1/2SLPI are within 0.88 Å in comparison with these X-ray structures. However, the side-chains of P3 Gln70i and P5 Tyr68i take various conformers in other complexes. For PPT, RMSD values by superposing of main-chain atoms between three PPT molecules in an asymmetric unit and those of already reported PPT structures [PDB codes 1s6h (Thomas *et al.*, 2004 ▶), 1s85 (Thomas *et al.*, 2004 ▶), 1z7k (Ibrahim & Pattabhi, 2004 ▶), 2a31 (Transue *et al.*, 2006 ▶), 1h9h (Krätzner *et al.*, 2005 ▶), 1tx6 (Park *et al.*, 2004 ▶), 1tfx (Burgering *et al.*, 1997 ▶)] were within 0.51 Å. They have almost the same main-chain structures.

**Figure 2:**
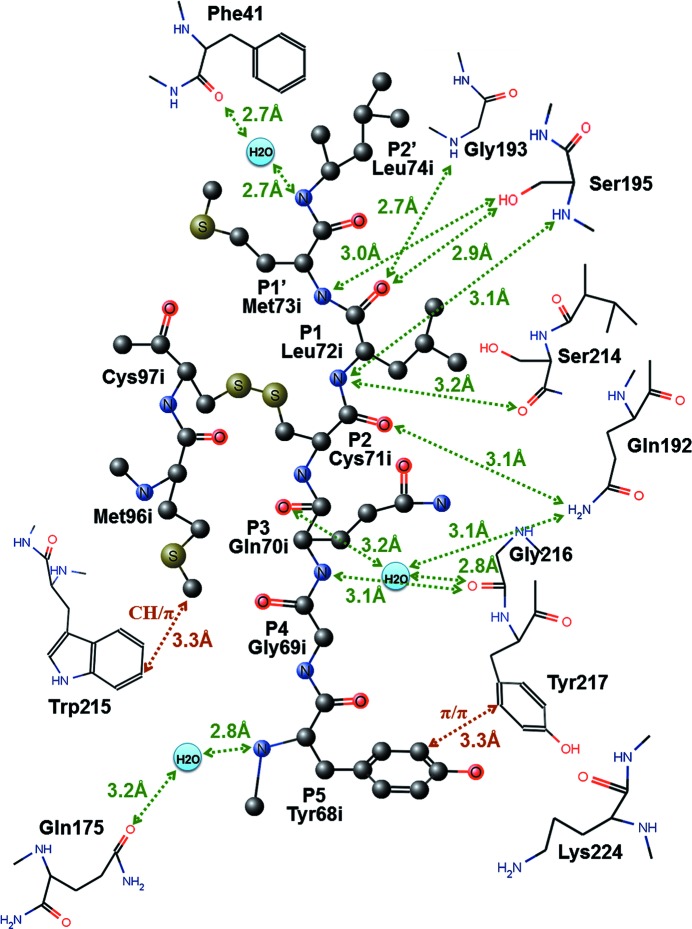
Schematic representation of the hydrogen bonding and the hydrophobic interactions between 1/2SLPI and PPT.

The intermolecular hydrogen-bonding modes between the binding regions of 1/2SLPI and PPT are shown in Fig. 2 ▶. These intermolecular hydrogen-bonding patterns between main-chains of 1/2SLPI and PPT are very similar to the 1/2SLPI–HNE and SLPI–chymotrypsin complexes. The differences between three complexes with 1/2SLPI are found in the interaction in the P5 Tyr68i residue and the secondary binding region (Met96i). The primary binding region appears to range from P3 Gln70i to P2′ Leu74i by intermolecular hydrogen bonds (green dotted arrows in Fig. 2 ▶). An intermolecular interaction between P5 Tyr68i CD1 and Tyr217 CG1 should be added to the primary binding region too. Met96i is also located close to Trp215 with a slightly longer distance than the usual van der Waals contact as the secondary binding site. In all complex structures (1/2SLPI–PPT, 1/2SLPI–HNE and SLPI–chymotrypsin), the Leu72i residue of 1/2SLPI is located in the S1 pocket as a P1 residue. A main part of the affinity and inhibitory activity of 1/2SLPI also depends on the P1 residue which penetrates deeply into the S1 pocket of the proteases.

**Figure 3:**
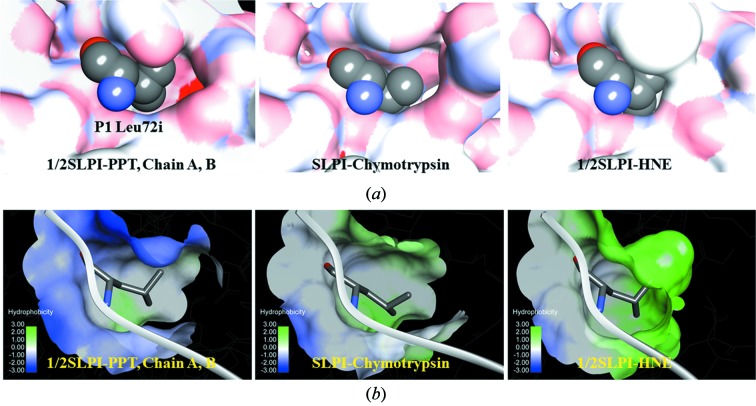
(*a*) Electrostatic surface maps of S1 pockets. (*b*) Hydrophobicity in S1 pockets.

A comparison of the S1 pockets of PPT, chymotrypsin and HNE with P1 Leu72i of 1/2SLPI is shown in Fig. 3(*a*) ▶. A most important point emerged in this crystallographic study that 1/2SLPI has the possibility to bind the S1 pocket of trypsin with the P1 Leu72i side-chain. The X-ray crystal structures of many serine protease inhibitors in complex with PPT, chymotrypsin, HNE and others have been determined. However, as yet, any protease inhibitor and its variants with the P1 Leu residue have not crystallized with trypsin. 1/2SLPI is able to inhibit trypsin in several n*M* and to be crystalized with PPT in spite of the P1 Leu72i residue inhibitor. Fig. 3(*b*) ▶ shows the hydrophobicity of each S1 pocket of 1/2SLPI with PPT, chymotrypsin and HNE. Even in complex with PPT, the conformations of P1 Leu72i of SLPI are quite similar to the complexes of 1/2SLPI–HNE and SLPI–chymotrypsin. The hydrophobic interaction also seems to be the main interaction in Fig. 3(*b*) ▶.

**Figure 4:**
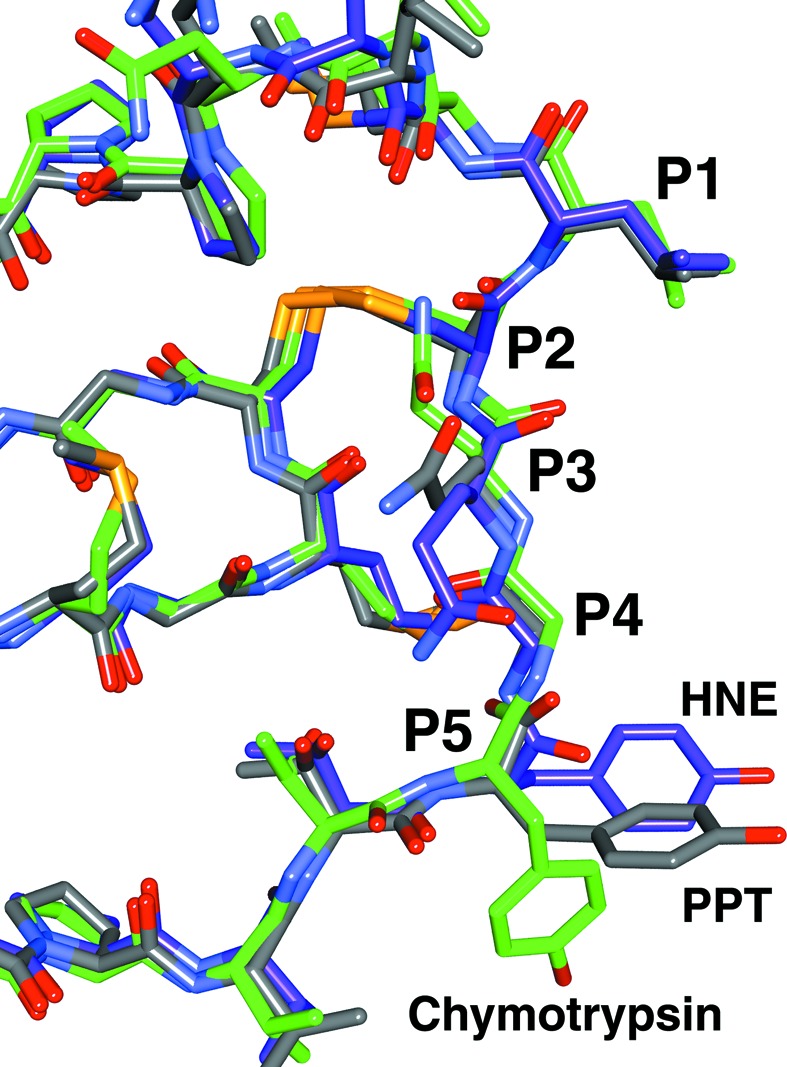
Superposition of 1/2SLPI molecules bound to PPT, chymotrypsin and HNE.

In the 1/2SLPI–PPT complex, the S5 pocket of PPT formed by residues Tyr217 and Lys224 interacts with the P5 Tyr68i residue of 1/2SLPI. Fig. 4 ▶ shows the superposition of three 1/2SLPI molecules which bind to three enzymes. The side-chain of the P5 Tyr68i residue takes different conformations. As the enzymes have not drastically changed the structure of the S5 site by 1/2SLPI binding, 1/2SLPI should change the P5 Tyr68i conformation to accommodate each S5 pocket. This flexibility causes the inhibitory mechanism as 1/2SLPI can inhibit various serine proteases broadly. Additionally, energy evaluations of the inhibitory mechanism of 1/2SLPI from structural results are consistent such that IFIE (inter-fragment interaction energy) values from FMO (an *ab initio* fragment molecular orbital) calculations are negative values equivalent to P1, P1′ and P5 in the 1/2SLPI–PPT complex (Table 3 ▶). All FMO calculations were performed using the *ABINET-MP* programs (http://www.ciss.iis.u-tokyo.ac.jp/rss21/index.html). A detailed FMO study will be presented elsewhere.

In conclusion, our study shows the first structural result of the interaction mode between the biological protease inhibitor with the P1 Leu residue and trypsin. Unique and characteristic inhibitory mechanisms of 1/2SLPI are found from comparisons with three co-crystal structures (1/2SLPI–PPT, 1/2SLPI–HNE and SLPI–chymotrypsin). 1/2SLPI controls the inhibitory activity by using moieties from P2′ to P5 and can be adapted to various proteases flexibly. In the evolution process, the C-terminal domain of SLPI must play a multiple inhibitor to protect the human body from urgent inflammations and must use the residues of the wide primary contact region (from P2′ up to P5) to prevent diseases effectively. This endogenous serine protease inhibitory mechanism of 1/2SLPI gives a hint of designing drugs of middle-size inhibitors with specific multi-target profiles (Besnard *et al.*, 2012 ▶) of the next generation which can inhibit various targets effectively and safely.

## Supplementary Material

PDB reference: 4doq

## Figures and Tables

**Table 1:** Crystallographic data for the 1/2SLPI–PPT complex Values in parentheses are for the highest-resolution shell (2.05–2.00 Å).

Data collection	
Space group	*P*2_1_
Unit-cell parameters	
*a* (Å)	40.5
*b* (Å)	118.6
*c* (Å)	93.4
α = γ (°)	90
β (°)	90.7
Resolution (Å)	50.06–2.00 (2.05–2.00)
*R*_merge_ (%)	5.4 (25.7)
*I*/σ(*I*)	12.2 (4.3)
Completeness (%)	96.8 (96.3)
Multiplicity	3.48 (3.45)
Number of reflections	200155
Number of unique reflections	57597

Refinement statistics	
Reflections used for refinement	54686
*R*_cryst_/*R*_free_ (%)	18.2/24.2
No. of atoms	
Total	6168
Protein	5568
Heterogen	88
Water	512
*B*-factor (Å^2^)	30.2
R.m.s. deviations	
Bond distances (Å)	0.018
Bond angles (°)	1.701

**Table 2:** *K*_i_ values of 1/2SLPI against serine proteases

	*K*_i_ (n*M*)
HNE	0.65
Bovin α-chymotrypsin	0.87
BPT	36
PPT	140
PPE	680

**Table 3:** IFIE values of each P5–P2′ residue against overall PPT, chymotrypsin and HNE

	FMO calculated IFIE values (kcal mol^−1^)		
1/2SLPI seq	For PPT	For chymotrypsin	For HNE
P2′ Leu74i	−16.9	−12.1	−10.7
P1′ Met73i	−45.4	−26.1	−40.4
P1 Leu72i	−40.9	−20.4	−37.9
P2 Cys71i	−24.5	−14.4	−11.5
P3 Gln70i	−19.8	−10.7	−11.5
P4 Gly69i	−15.7	−44.9	−26.5
P5 Tyr68i	−38.0	−4.7	−16.6
